# Clinical and Genetic Characteristics of *ABCC8* Nonneonatal Diabetes Mellitus: A Systematic Review

**DOI:** 10.1155/2021/9479268

**Published:** 2021-09-30

**Authors:** Meng Li, Xueyao Han, Linong Ji

**Affiliations:** Department of Endocrinology and Metabolism, Peking University People's Hospital, Peking University Diabetes Center, Beijing, China 100044

## Abstract

**Objectives:**

Diabetes mellitus (DM) is a major chronic metabolic disease in the world, and the prevalence has been increasing rapidly in recent years. The channel of K_ATP_ plays an important role in the regulation of insulin secretion. The variants in *ABCC8* gene encoding the SUR1 subunit of K_ATP_ could cause a variety of phenotypes, including neonatal diabetes mellitus (*ABCC8-*NDM) and *ABCC8*-induced nonneonatal diabetes mellitus (*ABCC8*-NNDM). Since the features of *ABCC8*-NNDM have not been elucidated, this study is aimed at concluding the genetic features and clinical characteristics.

**Methods:**

We comprehensively reviewed the literature associated with *ABCC8*-NNDM in the following databases: MEDLINE, PubMed, and Web of Science to investigate the features of *ABCC8*-NNDM.

**Results:**

Based on a comprehensive literature search, we found that 87 probands with *ABCC8*-NNDM carried 71 *ABCC8* genetic variant alleles, 24% of whom carried inactivating variants, 24% carried activating variants, and the remaining 52% carried activating or inactivating variants. Nine of these variants were confirmed to be activating or inactivating through functional studies, while four variants (p.R370S, p.E1506K, p.R1418H, and p.R1420H) were confirmed to be inactivating. The phenotypes of *ABCC8*-NNDM were variable and could also present with early hyperinsulinemia followed by reduced insulin secretion, progressing to diabetes later. They had a relatively high risk of microvascular complications and low prevalence of nervous disease, which is different from *ABCC8-*NDM.

**Conclusions:**

Genetic testing is essential for proper diagnosis and appropriate treatment for patients with *ABCC8*-NNDM. And further studies are required to determine the complex mechanism of the variants of *ABCC8*-NNDM.

## 1. Introduction

Diabetes mellitus (DM) is a major chronic metabolic disease in the world, and its prevalence has increased rapidly in recent years. Genetic and environmental conditions contribute to DM. The type of monogenic diabetes is the main etiology for diabetes. Maturity-onset diabetes of the young (MODY) is a kind of monogenic diabetes characterized by autosomal dominant inheritance. It is reported that the prevalence of MODY is 1 ~ 5% [1]. The activating variants of *ABCC8* also could cause MODY. The *ABCC8* gene encoding sulfonylurea receptor (SUR), which is the regulatory subunit of K_ATP_ channel, plays a key role in regulating insulin secretion [2, 3]. K_ATP_ channel is a hetero-octamer and consists of four inwardly rectifying proteins of the potassium channel Kir6.2 and four regulatory subunits of the sulfonylurea receptors [4, 5]. The enhanced glucose metabolism results in a change of ADP/ATP and leads to the closure of the K_ATP_ channel, which in turn induces membrane depolarization and triggers the opening of the voltage-dependent Ca^2+^ channel, which stimulates the release of insulin [6, 7]. Besides, variants in *ABCC8* gene could cause hyperinsulinemic hypoglycemia (HH) due to inactivating variants which have an impaired response to magnesium adenosine diphosphate- (MgADP-) mediated opening of the channel [6, 8, 9]. Therefore, variants in *ABCC8* gene could cause variable phenotypes: diabetes and HH, due to the different effects of the variants on channel function [9–13]. According to the onset age, DM induced by the *ABCC8* variants are classified as two major groups of disorders—*ABCC8*-induced nonneonatal diabetes mellitus (*ABCC8*-NNDM) and *ABCC8-*induced neonatal diabetes mellitus *ABCC8*-NDM. Although the features of *ABCC8*-NDM have been well evaluated, the studies on the clinical and genetic features of *ABCC8*-NNDM were limited. And these studies were mainly conducted in Europe and America. Bowman et al. first identified *ABCC8* missense variants as a cause of MODY by testing sulfonylurea-sensitive *HNF1A* and *HNF4A* variant-negative MODY cases with no family history of neonatal diabetes [10]. Then, Johansson et al. identified a patient with *ABCC8-*MODY by exome sequencing in an analysis of variant-negative MODY cases by Sanger sequencing [14]. Additionally, potential pathogenic alterations in the *ABCC8* gene were also identified in genetic studies. It has been shown that the prevalence of *ABCC8* variants was estimated to be 0.5 ~ 1.5% in different cohorts [15–17]. However, the clinical phenotype has not been well established. In addition, the development in the field of *ABCC8* gene-related diabetes has included de novo variants identified by new rapid molecular genetic features, symptoms, and medical therapy (sulfonylureas, DPP4-inhibitor).

Therefore, we systematically reviewed the literature related to *ABCC8*-NDM and *ABCC8*-NNDM to comprehensively conclude the genetic and clinical features of *ABCC8*-NNDM. The review article has summarized the updated advance of *ABCC8*-NNDM and included de novo variants, clinical symptoms, and medical therapy.

## 2. Materials and Methods

### 2.1. Study Subjects

A total of 144 patients with *ABCC8*-NNDM were included to analyze the clinical and genetic features in previous literature. The literature search has been conducted until Sep. 2020. We systematically identified all potentially relevant articles from the following three electronic databases: MEDLINE, PubMed, and Web of Science. Search terms about diabetes—such as “maturity-onset diabetes of the young (MODY),” “Neonatal diabetes mellitus,” “Neonatal diabetes” and “*ABCC8*-MODY,” and Sulfonylurea receptor 1, for example, “Sulfonylurea receptor 1,” “*ABCC8*,” and “KATP channels”—were used in various combinations and permutations across the databases. Language restriction (English) was applied. The criteria for inclusion were patients with *ABCC8*-NNDM and those with *ABCC8*-NDM in previous studies. We systematically reviewed the related studies, including population-based studies, reviews, functional studies, and meta-analysis. The criteria for exclusion were repetitive literature and unavailable data. The genetic information of *ABCC8* gene was as follows: accession number: NM_000352.4, NP_000343.2.

For data extraction, clinical information, including demographics, initial presentation, treatment of diabetes, physical examination results, laboratory test results, and information of genetic variants of the patients, was extracted.

### 2.2. Classification of the Pathogenicity of *ABCC8* Variants

The pathogenicity of the variants was classified according to the established guidelines of the American College of Medical Genetics and Genomics and the Association for Molecular Pathology (ACMG-AMP) [18]. We classified these variants into the following categories: pathogenic, likely pathogenic, uncertain significance, likely benign, and benign. We used two or more lines of computational evidence (PROVEAN (http://provean.jcvi.org), SIFT (http://sift.jcvi.org/), Polyphen2 (http://genetics.bwh.harvard.edu/pph2/index.shtml), and MutationTaster (http://mutationtaster.org)) to support a deleterious effect on the gene for pathogenic supporting 3 (PP3) according to the guidelines of the ACMG-AMP. According to the guidelines, each pathogenic criterion is weighted as very strong (PVS1), strong (PS1–4), moderate (PM1–6), or supporting (PP1–5).

### 2.3. Conservation of the Variants

We conducted multiple sequence alignment (MSA) to align sequences of *ABCC8* protein from a few vertebrate species by ClustalW server (https://www.genome.jp/tools-bin/clustalw) to interpret the conservation of these sequences. The result of MSA from ClustalW was plotted using ESPript (Easy Sequencing in Postscript 3.0, http://espript.ibcp.fr) [19, 20]. The species and GenBank accession numbers of the *ABCC8* sequences adopted were the following: *Homo sapiens*—NP_000343.2, *Callithrix jacchus*—XP_035121815.1, *Chlorocebus sabaeus*—XP_008003585.1, *Danio rerio*—NP_001166118.2, *Sus scrofa*—XP_008003585.1, and *Vulpes vulpes*—XP_025863953.1. We followed the methods of Li et al. [21].

### 2.4. Statistical Analysis

Normally distributed variables were expressed as mean ± SD, and they were compared using *t*-tests. Categorical variables were presented as numbers and percentages. A Chi-square was adopted for categorical data. Analyses were performed using SPSS version 23.0.

## 3. Results

### 3.1. The Clinical and Genetic Characteristics of Patients with *ABCC8*-NDM Described in Previous Studie*s*

We have systematically reviewed the literature reporting variants in *ABCC8*-NDM. 175 probands with *ABCC8*-NDM (including 139 patients with heterozygous variants, 21 patients with homozygous variants, one patient with a mosaic variant, and 14 patients with compound heterozygous variants) variants were found owing to 110 *ABCC8* (Table 1). Among those probands, 66 patients were reported as having transient neonatal diabetes mellitus, 92 as having permanent neonatal diabetes, and 17 as having an unknown type of diabetes due to a lack of follow-up. These variants caused *ABCC8*-NDM with either a dominant or recessive genetic pattern and were scattered throughout the functional regions of the gene (Table 1 and Supplementary Figure 1).

All these patients presented with impaired insulin secretion, and 18 of the 110 variants were confirmed to be activating in functional studies and affect the channel inhibition by different molecular mechanisms. Then, those variants led to impaired insulin secretion and diabetes, as shown in Table 1.

The birth weight of 99 probands was available. Thirty-two probands (32%) had a birth weight < 2,500 g, and only one proband (1%) had a birth weight of >4,000 g. Forty-three of the 175 (24.6%) probands with *ABCC8*-NDM had neurological manifestations. In addition, 21 (12.0%) patients had developmental delay and epilepsy syndrome (DEND), 5 (2.9%) patients had intermediate DEND syndrome, 5 (2.9%) had seizures, and 12 (6.9%) had other neurological symptoms. This was similar to the previous study reporting approximately 20% of patients with K_ATP_ channel variants developed neurological symptoms [22]. The variants reportedly associated with the neurological phenotype were across all functional regions of the *ABCC8* gene.

### 3.2. The Genetic Characteristics of Patients with *ABCC8*-NNDM Reported in Previous Studies

After systematical reviewing the literature related to the *ABCC8*-NNDM studies, 87 probands were identified with 71 *ABCC8* genetic variant alleles, including 75 patients with heterozygous variants, four with homozygous variants, and eight with compound heterozygous variants (Table 2, Supplementary Table 1, Supplementary Figure 1). The domains where the variants are located have been displayed in Table 2. By available data and bioinformatics analysis, 47 and 15 variants of the 71 variant alleles were classified as likely pathogenic and pathogenic, respectively, while nine variants were of uncertain significance (VUS) (Supplementary Table 2).

Nine (including p.Y356C, p.R370S, p.L582V, p.R825W, p.R1182Q, p.P1198L, p.R1418H, p.R1420H, and p.E1506K) of 71 genetic variant alleles were confirmed to be activating or inactivating through functional studies (Table 2). Among them, five activating variants (p.Y356C, p.L582V, p.R825W, p.R1182Q, and p.P1198L) have been demonstrated that channel inhibition by ATP was reduced and less insulin was secreted [15, 23–26]. The remaining four inactivating variants (p.R370S, p.E1506K, p.R1418H, and p.R1420H) were found to decrease K_ATP_ channel activity and bring about diabetes [27–30]. The patients with inactivating variants had hyperinsulinemic hypoglycemia in early life and progressed to diabetes later.

**Figure 1:**
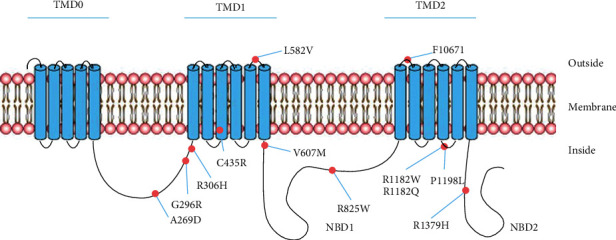
A schematic of the transmembrane topology of SUR1 showing the location of the variants both in *ABCC8*-NDM and *ABCC8*-NNDM. The transmembrane domains (TMD) include TMD0, TMD1, and TMD2. The nucleotide-binding domains (NBD) are indicated by NBD1 and NBD2, and the cytosolic linker L0 is between TMD0 and TMD1. *ABCC8*-NDM: *ABCC8*-induced neonatal diabetes mellitus; *ABCC8*-NNDM: *ABCC8*-induced nonneonatal diabetes mellitus.

In addition, twelve variants (including p.A269D, p.G296R, p.R306H, p.C435R, p.L582V, p.V607M, p.R825W, p.R1182W, p.R1182Q, p.P1198L, p.F1067I, and p.R1379H) of *ABCC8* were reported both in patients with *ABCC8-*NDM and in those with *ABCC8-*NNDM (Table 2 and Figure 1). The above variants were located in the domains of the L0 linker region (L0), transmembrane domain 1 (TMD1), nucleotide-binding domain 1 (NBD1), transmembrane domain 2 (TMD2), and nucleotide-binding domain 2 (NBD2) (Figure 1). The same variant could cause different onset ages of diabetes.

#### 3.2.1. Evolutionary Conservation of Sites of Variants Both in Patients with ABCC8-NDM and in Those Patients with ABCC8-NNDM

**Figure 2:**
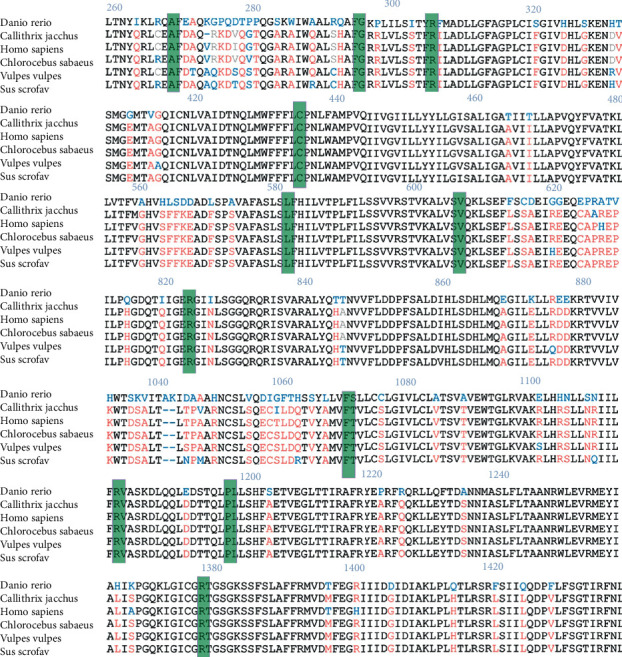
Multiple sequence alignment of the *ABCC8* gene. Multiple sequence alignment of *ABCC8* of a vertebrate species including *Homo sapiens* was analyzed. The black font represents strictly conserved amino acid residues, while sites with sequence identities of 70% or more are in red. Twelve variants identified through this study are highlighted in green.

The conservation analysis was carried out using ClustalW and ESPript 3.0 tools. Multiple sequence alignments of *ABCC8* in the vertebrate species were selected for this analysis to show the sequence conservation of amino acid residues between them (Figure 2). It has been demonstrated that the amino acid residues of these twelve variants of *ABCC8* both in *ABCC8-NDM* and *ABCC8-NNDM* in the literature were conserved across the organisms queried.

#### 3.2.2. ABCC8-NNDM due to Gain-of-Function and Loss-of-Function of Variants

Previous studies reported that both gain-of-function and loss-of-function variants in *ABCC8* could cause diabetes. The first loss-of-function *ABCC8* variant was a heterozygous inactivating *ABCC8* p.E1506K variant, which presented with HH, followed by glucose intolerance and diabetes in later life [31]. This distinct phenotype was demonstrated in a mouse model carrying the p.E1506K variant of *ABCC8* [28] and in patients carrying other rare variants of *ABCC8*, such as p.R370S, p.R1418H, and p.R1420H [29, 30, 32]. Two Japanese probands with hypoglycemia in infancy due to heterozygous inactivating variants progressing to hyperglycemia were also reported [33, 34]. Therefore, the subtype of *ABCC8*-NNDM due to inactivating variants could implicate the etiology of diabetes.

Among the 87 probands previously reported, 24% (21/87) carried inactivating *ABCC8* variants reported in hyperinsulinemia, whereas 24% (21/87) carried activating *ABCC8* variants were also associated with NDM. And the remaining 52% (45/87) carried variants with undetermined molecular mechanism. In the previously reported cases of *ABCC8*-NNDM, it was estimated to be ~25% patients with activating *ABCC8* variants and ~25% with inactivating variants. The molecular mechanisms of the remaining ~50% variants were needed further investigation.

#### 3.2.3. The Clinical Characteristics of Patients with ABCC8-NNDM Reported in Previous Studies

A total of 144 patients with *ABCC8* variants including the probands and their hyperglycemic relatives (125 Caucasians, 15 East Asians, three Africans, and one Chinese) were analyzed. The clinical and genetic characteristics of them are shown in Table 2 and Supplementary Table 1. The diagnosed age was reported in 71 probands. Among them, three (4%) were diagnosed with diabetes when <6 years old, 28 (39%) when 6–18 years old, and 40 (56%) when 18–40 years old. According to their body mass index (BMI) at diabetes onset, 10 (27%) of the 37 patients (seven probands and three relatives) who were diagnosed when ≥18 years old were overweight or obese. According to the available data, 30 patients were described using sulfonylureas (SUs) for glucose control. 22/30 (73.3%) patients have shown to be effective with SUs, while the levels of HbA1c were less than 7.0%. We found that just three probands with *ABCC8* variants, including compound variants of p.H103Y and p.R74Q and missense variants of p.A1457T and p.E1506K, had microalbuminuria [35–37]. Reilly et al. have described that retinopathy was also common microvascular complication and that 5 out 10 patients with *ABCC*8 variants had diabetic retinopathy [37]. A similar case was reported by Ovsyannikova et al. [36], where the patient was diagnosed with diabetes at age 27 years (p.A1457T variant in *ABCC8*), and during the initial investigation, he had nonproliferative retinopathy and a raised microalbumin creatinine ratio.

As is known to all, neurological features are essential for *ABCC8*-NDM, and forty-three (24.6%) probands had neurological manifestations among the 175 reported probands with *ABCC8*-NDM according to the published literature. In the *ABCC8*-NNDM group, just one proband with the variant of p.A1457T had epilepsy independent of hypoglycemia [36], and two probands with the variants of p.R1418H and p.R1420H had seizure due to hypoglycemia [29, 30]. Compared with the *ABCC8-*NDM group, the frequency of the neurological phenotype in the *ABCC8*-NNDM group was significantly lower (1/87 (1%) vs. 43/175 (24.6%), *P* <0.001), and we did not include neurological features due to hypoglycemia.

We have descripted above that there were 24% probands carrying activating variants, 24% carried inactivating variants, and 52% carrying undetermined variants among the 87 probands. Based on the available data, we further compared the clinical features between the probands with activating and inactivating variants. There was no significant difference in diagnosed age and BMI between the two groups (diagnosed age: 28.9 ± 11.6, *n* = 14 vs. 19.1 ± 10.0, *n* = 0.981, *P* = 0.960; diagnosed BMI: 22.5 ± 3.3, *n* = 6 vs. 22.5 ± 4.3, *n* = 9, *P* = 0.981). Among the inactivating group, two probands with the variants of p.R1418H and p.R1420H had seizures due to hypoglycemia [29, 30], while no probands were reported with neurological symptoms among the activating group. And one proband with inactivating p.E1506K variant had microalbuminuria [37]. In addition, the prevalence of hyperinsulinemia and hypoglycemia was significantly higher in the inactivating group than the activating group (13/21 (61.9%) vs. 0/21 (0.0%), *P* < 0.001).

## 4. Discussion

To the best of our knowledge, it is the first time for our study to systematically review the literature and comprehensively investigate the genetic and clinical features of *ABCC8*-NNDM. From the previous studies, we identified 144 patients with *ABCC8*–NNDM and found that ~25% and ~25% of the previously reported *ABCC8*-NNDM cases had activating and inactivating *ABCC8* variants, while the remaining ~50% had uncertain functional variants. These patients had relatively successful glucose control after the treatment of SUs and might have a relative high risk of diabetic microvascular complications.

As is known to all, gain-of-function variants in the *ABCC8* gene are one of the main causes of NDM. With the development of genetic analysis, the *ABCC8* variants in NNDM were also reported. Many potential pathogenic alterations were also identified in *ABCC8*. A study performed in a French adult type 2 diabetic outpatient cohort with 139 patients identified two (1.5%) likely causative variants in *ABCC8* [15]. Another study in a large cohort of nonobese patients with diagnosed age < 40 years and a family history of diabetes found 8 (8/1564, 0.5%) *ABCC8* variants [16]. In addition, an East Asian study found one (0.9%) *ABCC8* variant among 109 suspected monogenic diabetes patients [17]. The prevalence of *ABCC8* variants was estimated to be 0.5 ~ 1.5% in different studies. It suggests that the subunit K_ATP_ channel of SUR1 encoding the *ABCC8* gene is responsible for a small subset of NNDM.

The *ABCC8* gene encodes SUR subunit of K_ATP_ channels, which links cell metabolism to electrical activity by regulating potassium movement across the membrane [38]. Closure of the channel as a result of ATP-binding led to *β*-cell membrane depolarization and opening of voltage-dependent and calcium-channels and calcium mediated release of insulin. Activating variants in the *ABCC8* gene led to an increased probability of opening of the potassium channel, therefore preventing any activation of the voltage-dependent calcium channel and any glucose-induced insulin secretion [39], leading to NDM, early onset diabetes, and MODY [10, 40]. In previous studies, there are 18 variants that were confirmed to be activating by functional studies. However, the pathophysiology of inactivating variants in *ABCC8* means that lack of functional K_ATP_ channels leads to depolarized *β*-cells and elevation of cytosolic calcium, which result in continuous insulin secretion and independent of plasma glucose concentration [6, 8]. In addition, 21 (24%) probands with dominant loss-of-function variants in *ABCC8* previously reported could cause hyperinsulinism in the early period and progress to diabetes later. Four inactivating variants (p.R370S, p.E1506K, p.R1418H, and p.R1420H) have been demonstrated to decrease K_ATP_ channel activity and dysregulation of insulin secretion in functional studies, with the consequence that patients with these variants progressed to diabetes in later life. As a consequence, both activating and dominantly inactivating variants have been considered the key cause of *ABCC8*-NNDM.

The underlying mechanism by which loss-of-function variants of *ABCC8* subsequently cause the remission of HH and future hyperglycemia is complex and required to be elucidated. Recently, basal insulin secretion was observed to be elevated in human islets with inactivating *ABCC8* variants, but insulin secretion response to glucose was impaired [41]. And apoptotic beta cells increased in transgenic mice with inactivating K_ATP_ channels [42, 43]. Besides, insulin content and gene expression decreased and led to the disruption of insulin secretion and glucose intolerance in the mouse model with an inactivating variant of *ABCC8* [28]. The above factors could partly address the mechanism by which K_ATP_ defects cause diabetes in patients with loss-of-function variants. To date, there have been far-from adequate functional studies to elucidate the exact impact of the different variants. Further studies are still needed to account for the complex underlying mechanisms resulting in the remarkable phenotypic heterogeneity related to inactivating *ABCC8* variants.

Great importance is to be attached to the diabetic complications. It could be seen that just three probands with variants were reported to have diabetic kidney injury. It is uncertain whether patients with *ABCC8-*NNDM had a higher risk of diabetic kidney disease (DKD). Mice with the homozygous *ABCC8* p.E1506K^+/+^ variant (an inactivating variant), presenting with hyperinsulinemia early in life followed by diabetes and early DKD later in life, were observed [28, 44]. In the mouse model, glucose could induce histone modifications, which drove the expression of proinflammatory genes and thereby predisposed to diabetic kidney disease. Further studies are also required to confirm the risk and the precise diabetic kidney disease mechanism of *ABCC8* variants. Besides, the frequency of diabetic retinopathy was ~50 percent among the patients with *ABCC8* variants. Although dyslipidaemia, hypertension, and possible genetic factors contribute to the early manifestation of diabetes complications, the *ABCC8* variants may be responsible for the rapid progression to proliferative retinopathy. SUR1 is also expressed in the retinal vessels, and glibenclamide could inhibit adenosine-induced retinal vasodilation [45, 46], which occurs by interacting with K_ATP_ channels in retinal vessel pericytes. In models of postinfarct central nervous system oedema, the SUR1 expression has been observed to upregulate in injured nervous tissue, and inhibiting SUR1-induced ion channel modulation with the drug glibenclamide could protect the central nervous system from ischaemia-reperfusion and traumatic brain injury [47].

As we known, neurological features are important features for *ABCC8*-NDM. From the published literature, we found that sixty (34%) probands had neurological manifestations among 175 reported probands with *ABCC8*-NDM. K_ATP_ channels are predominately expressed in endocrine tissues such as the pancreatic islet cells and the nervous system. The deleterious effect on the nervous system of K_ATP_ channel activating variants is likely related to the neurological features, including more severe DEND and iDEND [48, 49]. The reported variants associated with a neurological phenotype were distributed across all functional regions of the gene, while only one patient had a neurological manifestation independent of hypoglycemia (1%) among patients with *ABCC8*-NNDM [36]. Compared to the *ABCC8-*NDM group, the frequency of a neurological phenotype in the *ABCC8*-NNDM group was significantly lower. Activating variants reduce the ability of ATP to inhibit ion channel activity, thus increasing the magnitude of the K_ATP_ current, which hyperpolarizes brain and muscle cells and accounts for the neurological phenotype [39, 50, 51].

According to the previous published studies reporting SUs on the treatment of *ABCC8* variants induced diabetes, we found that 73.3% of the patients owing to *ABCC8* variants with SUs got successful glucose control. As the widely used drugs for the treatment of patients with type 2 diabetes, SUs could bind specifically to the SUR1 subunit, then closing the K_ATP_ channel via an ATP-independent mechanism and therefore increasing the insulin secretion of *β* cells [52]. Several studies observed that patients with NDM were transferred to SUs successfully after molecular genetic diagnosis of *ABCC8* variants [53, 54]. Up to 90–95% of patients with NDM due to using *ABCC8* and KCNJ11 variants are able to be taken off of insulin therapy after initiation of SUs therapy [55, 56]. A recent meta-analysis also showed the estimated success rate was 90.1% in the SU treatment for *ABCC8*-NDM [57]. Therefore, SUs are effective for diabetic patients due to activating *ABCC8* variants. However, due to different types of variants and variable clinical phenotypes, the correct treatment may be different. In addition, the sensitivity to SUs was variable in patients with *ABCC8* variants. As majority, but not all, patients were successful to transfer from insulin to SUs [58]. In addition, two Japanese patients with hypoglycemia in infancy progressed to diabetes later in life due to the *ABCC8* heterozygous inactivating variants and got better glucose control treated with DPP4 inhibitors [33, 34]. It might be useful for patients with inactivating variants to be treated with incretin-related drugs. We need to consider the genetic features and the response of treatment to facilitate individualized therapy.

Although we systematically reviewed the previous studies on *ABCC8*-NNDM, the sample size was limited. More studies are needed to better summarize its characteristics. In addition, we found that a few patients in the case reports were effective for new hypoglycemic drugs, but there was a lack of randomized controlled trials and longitudinal follow-up studies to help us determine the long-term efficacy and the impact on complications and neuropathy. Although we used the ACMG guideline to interpret the pathogenicity of *ABCC8* variants, the precise molecule mechanisms are still needed to clarify in vivo and vitro studies.

## 5. Conclusion

Our study comprehensively concluded the genetic and clinical features of *ABCC8*-NNDM. The variants of *ABCC8*-NNDM consist of activating and inactivating ones. The phenotypes of these patients varied with good effect for SUs and had a risk of diabetic complications. It is also essential to make a precise genetic diagnosis for appropriate treatment of them to reduce episodes of hypoglycemia and diabetic complications. Next generation sequencing (NGS) enables a rapid and cost-effective diagnosis, and it should be taken into consideration for the *ABCC8* gene in early onset diabetes. In the future, studies are needed to account for the mechanisms resulting in the remarkable phenotypic heterogeneity related to *ABCC8* variants.

## Figures and Tables

**Table 1:** Variants of *ABCC8* causing neonatal diabetes mellitus reported in previous studies.

Topological domain	Variant (protein effect)	Zygosity	Neurological features	Reference
TMD0	p.S8R, p.V86A, p.V86G, p.A90V, p.F132V, p.L135P	Het		[50, 58–63]
	p.I49F, p.F132L^∗^	Het	DEND	[39, 50, 58, 59, 64–66]
	p.N72S	Mosaic		[50, 58, 59, 64, 67]

L0	p.E208K, p.D209E, p.D209N, p.Q211K, p.D212E, p.D212N, p.D212Y, p.R216C, p.L225P, p.T229N, p.R285Q, p.G296R	Het		[2, 50, 52, 58, 59, 61, 64, 66, 68–71]
	p.D212I	Het	Muscle hypotonia	[58, 59, 70]
	p.L213P, p.L213R^∗^, p.R306H	Het	DEND	[40, 50, 59, 72, 73]
	p.A269D	Het	Hypotonia	[2, 50]
	p.T229I	Hom		[50, 58, 59]
	p.E208K+ p.Y263D	CH	DEND	[58, 59, 64]

TMD1	p.V324M, p.A355T, p.E350D, p.I395F, p.H410Y, p.S459R, p.Q485H, p.F536L, p.F577L, p.I585T	Het		[2, 13, 50, 53, 59, 65, 73–78]
	p.D424V	Het	Seizure	[79]
	p.C435R^∗^, p.L451P, p.V587G	Het	DEND	[40, 50, 59, 80, 81]
	p.L582V^∗^	Het	Slow ideation	[2, 23, 40, 50, 59]
	p.E382K, p.E382V	Hom		[50, 59, 64, 69, 82]

NBD1	p.V607M, p.R653Q, p.R825W, p.G832C, p.G832D, p.H862Y, p.R877Q, p.D897V, p.E939K	Het		[24, 61, 62, 66, 75, 82–87]
	p.R825W^∗^	Het	iDEND	[2, 24, 50, 52, 54, 58, 59, 63, 68–70, 87]
	p.E747X, p.R825W	Hom		[62, 88]

TMD2	p.H1023Y^∗^, p.S1053N, p.F1176L, p.Q1178R^∗^, p.R1182Q^∗^, p.R1182W^∗^, p.F1181S, p.P1198L^∗^, p.G1255S	Het		[2, 12, 23, 25, 26, 40, 50, 52, 58, 59, 66, 70, 73, 85, 89–95]
	p.N1122D	Het	Seizure	[50, 60]
	p.F1067I	Hom		[96]
	p.H1023R^∗^	Hom		[97]
	p.F1163L	Hom	DEND	[69, 82, 98]
	p.A1184E	Hom	Muscle weakness and seizures	[50, 59, 64]

NBD2	p.R1313H, p.R1379S, p.I1424V^∗^, p.E1506Q^∗^, p.E1506D^∗^, p.E1506G^∗^, p.V1522M	Het		[2, 13, 26, 40, 50, 58, 59, 76, 89, 99]
	p.R1379H	Het	Hyperkinesia	[2, 50, 59, 70, 80]
	p.R1379C^∗^	Het	Minor dystonia	[23, 40, 50, 52, 59, 70, 76, 100]
	p.R1379L^∗^	Het	DEND	[50, 58, 59, 100]
	p.A1536P	Het	Motor delay	[101]

L0 + NBD1	p.V215I + V607M, p.L225P^∗^ + D879N	CH		[58, 102, 103]

L0 + NBD2	p.T229I+ p.V1522L	CH		[58, 59, 64]

L0 + TMD1	p.P207S+ p.Y179X	CH		[59, 64]

NBD1 + TMD0	p.E747X+ p.E128K	CH		[88]

NBD2 + TMD2	p.E1327K+ p.V1523A + T1043QfsX74	CH		[59, 64, 104]

TMD0 + L0	p.A30V^∗^ + p.G296R^∗^	CH		[105]

TMD0 + NBD1	p.N23H+ p.R825W	CH		[63]

TMD0 + NBD2	p.P45L+ p.G1400R	CH	Reduced consciousness, seizures	[58, 59, 64, 106]
	p.L147R + p.R1379C	CH		[107]

TMD0 + TMD2	p.R168C+ p.G1256S	CH		[108, 109]

TMD1 + TMD2	p.V324M + p.R1394L	CH	DEND	[65]
	p.L438F+ p.M1289V, p.I544T+ p.R1214W, p.N426S+ p.R1182Q	CH		[13, 59, 66]

TMD2 + L0	A1263V + I196N	CH		[52]

ATP-binding cassette transporter subfamily C member 8 (*ABCC8*) (accession number: NM_000352.4) has 17 transmembrane helices arranged in groups of five (N-terminal transmembrane domain (TMD0)), six (TMD1), and six (TMD2). Two large cytosolic loops follow TMD1 and TMD2 and contain the nucleotide-binding domains (NBDs, including NBD1 and NBD2) that are characteristic of ATP-binding cassette (ABC) proteins. The L0 linker region is located between the TMD0 and the TMD1 domains. *ABCC8*-NDM: *ABCC8* variant-induced neonatal diabetes mellitus; Het: heterozygous; Hom: homozygous; CH: compound het; DEND: developmental delay and epilepsy syndrome; i-DEND: intermediate DEND syndrome. ^∗^ indicates that the variant has been demonstrated to be activating in functional studies.

**Table 2:** Variants of *ABCC8* causing *ABCC8*-NNDM reported in previous studies.

Topological domain	Variant (protein effect)	Zygosity	Neurological features	Reference
TMD0	p.S53C, p.V84I, p.E100K	Het		[10, 110, 111]
	p.L171F	Hom		[112]

L0	p.P201S, p.A235T, p.A269D^#^, p.G296R^#^, p.R306C, p.R306H^#^	Het		[15, 16, 32, 111, 113, 114]

TMD1	p.A355T, p.Y356C^∗^, p.R370S^∗^, p.C418R, p.C435R^#^, p.Q485R, p.V563D, p.L582V^∗^^#^	Het		[10, 15, 23, 27, 111, 113, 115, 116]

NBD1	p.V607M^#^, p.R620C, p.G658V, p.D673N, p.N780S, p.R825Q, p.R825W^∗^^#^, p.G832S, p.Q833K, p.H862R, p.E970V, p.A1536T	Het		[15, 16, 37, 83, 87, 111, 113, 117–120]

TMD2	p.G1008S, p.K1022Q, p.L1147R, p.R1182W^#^, p.R1182Q^∗^^#^, p.P1198L^∗^^#^, p.E1205K, p.N1244D	Het		[10, 16, 111, 116, 118, 121]
	p.F1067I^#^	Hom		[96]

NBD2	p.R1352H, p.A1366T, p.R1379H^#^, p.K1384Q, p.S1385F, p.A1390V, p.L1430F, p.Q1458E, p.A1472T, p.G1478R, p.R1493G, p.M1504T, p.E1506K^∗^, p.A1507P, p.M1513T, p.V1523L, p.A1536V, p.R1538Q	Het		[1, 10, 14–17, 31, 33, 37, 113, 115, 116, 118, 122–127]
	p.A1457T	Het	Epilepsy	[36]
	p.R1418H^∗^, p.R1420H^∗^	Hom		[29, 30, 128]

TMD0	p.H103Y + p.R74Q	CH		[35]

L0	p.G214R + p.V222M	CH		[10]

NBD1	p.R933X + c.3992-9G > A, p.F793Sfs71 + c.4608+4A > G	CH		[120, 129]

TMD2	p.L1191LfsX1207 + p.R1250X	CH		[129]
	p.L1147R + p.R1250X	CH		[129]

NBD2	p.R1420H + F591fs604X	CH		[128]

ATP-binding cassette transporter subfamily C member 8 (*ABCC8*) (accession number: NM_000352.4) has 17 transmembrane helices arranged in groups of five (N-terminal transmembrane domain (TMD0)), six (TMD1), and six (TMD2). Two large cytosolic loops follow TMD1 and TMD2 and contain the nucleotide-binding domains (NBDs, including NBD1 and NBD2) that are characteristic of ATP-binding cassette (ABC) proteins. The L0 linker region is located between the TMD0 and the TMD1 domains. “Neurological features” excludes seizures caused by hypoglycemia. *ABCC8*-NNDM: *ABCC8* variant-induced nonneonatal diabetes mellitus; Het: heterozygous; Hom: homozygous; CH: compound het. ^∗^ indicates that the damaging effect of the variant has been demonstrated in functional studies. # indicates that the variants have been reported to cause *ABCC8-*NDM and *ABCC8-*NNDM.

## Data Availability

The data used to support the findings of this study were included within the article.

## Supplementary Materials

Supplementary Materials Supplement Table 1: the clinical and molecular genetic characteristics of the patients with *ABCC8*-NNDM in the previous studies. Supplement Figure 1: a diagram illustrates the inheritance of *ABCC8* variants in probands with neonatal diabetes mellitus and nonneonatal diabetes mellitus. Variants showed in red represent that the variants were identified both in neonatal diabetes mellitus and nonneonatal diabetes mellitus. Abbreviations: *ABCC8*-NDM: *ABCC8* variant-induced neonatal diabetes mellitus; *ABCC8*-NNDM: *ABCC8* variant-induced nonneonatal diabetes mellitus. STROBE statement—checklist of items that should be included in reports of cohort studies.
