# Supplementary material for: Clinical and Genetic Characteristics of ABCC8 Nonneonatal Diabetes Mellitus: A Systematic Review
Source: J Diabetes Res. 2021 Sep 30;2021:9479268. doi: 10.1155/2021/9479268 (PMC8497126; doi:10.1155/2021/9479268)
Supplement: Supplementary Materials — Supplement Table 1: the clinical and molecular genetic characteristics of the patients with ABCC8-NNDM in the previous studies. Supplement Figure 1: a diagram illustrates the inheritance of ABCC8 variants in probands with neonatal diabetes mellitus and nonneonatal diabetes mellitus. Variants showed in red represent that the variants were identified both in neonatal diabetes mellitus and nonneonatal diabetes mellitus. Abbreviations: ABCC8-NDM: ABCC8 variant-induced neonatal diabetes mellitus; ABCC8-NNDM: ABCC8 variant-induced nonneonatal diabetes mellitus. STROBE statement—checklist of items that should be included in reports of cohort studies. [file 9479268.f1.pdf]

Supplementary Tables and Figures

Supplement Table 1. The clinical and molecular genetic characteristics of the patients with *ABCC8*-NNDM in the previous studies

No. [#]	No. of the patient in the family	Variant	Variant type	Age at diagnosis (ys)	BMI at diagnosis (kg/m^2)	Age at recruitment (ys)	BMI at recruitment (kg/m^2)	Treatment at recruitment	HbA1c at recruitment (%)	FPG at recruitment (mmol/l)	FINS at recruitment (uU/ml)
1	1	p.N780S	het	20*	/	/	20.3	/	6.4	5.9	/
2	1	p.G1008S	het	25*	/	/	21.4	/	6.7	5.8	/
3	1	p.A1472T	het	25*	/	/	22.8	/	5.0	5.3	/
4	1	p.E970V	het	30*	/	/	27	/	5.1	6.4	/
5	1	p.K1022Q	het	10*	/	/	20.4	/	14.1	8.8	/
6	1	p.G296R	het	/	/	/	/	/	/	/	/
7	1	p.Y356C	het	/	/	/	/	/	/	/	/
8	1	p.R825W	het	/	/	/	/	/	/	/	/
9	1	p.R1379H	het	/	/	/	/	/	/	/	/
10	1	p.E1506K	het	/	/	/	/	/	/	/	/
11	1	p.R825Q	het	6	/	19	26	Insulin	/	/	/
12	1	p.R306Cs	het	/	/	/	/	OAD	/	/	/
12	2	p.R306Cs	het	/	/	/	/	/	/	/	/
13	1	p.E1506K	het	28	/	/	/	/	/	5.0	/
14	1	p.H103Y, p.R74Q	het	9	/	17	23.6	OAD, insulin	11.0	9.8	/
15	1	p.R1493G	het	30	/	/	30.3	OAD	6.0	/	/
16	1	p.A1457T	het	26	/	28	/	OAD	/	/	/
17	1	p.R1379H	het	11	/	19	/	OAD	/	/	/
17	2	p.R1379H	het	/	/	/	/	/	/	/	/
18	1	p.R1420H	hom	/	/	/	/	/	/	/	/
19	1	p.R1418H	hom	1.3	/	/	/	/	/	/	/

19	2	p.R1418H	hom	28	22.4	45	/	OAD	8.1	/	/
19	3	p.R1418H	het	/	/	/	21.6	/	/	/	/
20	1	p.R1379H	het	17	24	63	/	OAD	7.3	/	/
21	1	p.C435R	het	15	20	39	/	OAD	6.7	/	/
22	1	p.L582V	het	36	21	38	/	OAD	6.2	/	/
22	2	p.L582V	het	32	37	37	/	OAD	6.4	/	/
23	1	p.Y356C	het	39	26	74	/	OAD	6.5	/	/
23	2	p.Y356C	het	35	20	35	/	/	5.5	/	/
24	1	p.P201S	het	53	22	80	/	OAD	7.4	/	/
25	1	p.C418R	het	53	31	74	/	OAD	6.2	/	/
26	1	p.R620C	het	46	25	56	/	OAD	6.8	/	/
27	1	p.R825W	het	49	28	58	/	OAD	6.2	/	/
28	1	p.R1420H, p.F591fs604X	Compound het	11	21.6		/	OAD	/	/	/
28	2	p.R1420H	het	/	/	/	/	/	/	/	/
29	1	p.A1366T	het	25	29.9	38	/	OAD	/	/	/
30	1	p.R370S	het	10.5	30.2	/	/	/	/	12.2	/
31	1	p.R933X, c·3992-9G>A	Compound, het	22	26.1	31	/	/	7.1	6.1	8
32	1	L1191LfsX1207, p.R1250X	het	10	26.2	17	/	/	5.2	5.8	15
33	1	p.L1147R, p.R1250X	het	16	22	24	/	/	5.0	5.1	9.3
34	1	p.R1379H	het	19	/	27	19.3	OAD	5.9	/	/
35	1	p.R1379H	het	11	/	19	18	OAD	9.5	/	/
36	1	p.G214R, p.V222M	het	15	/	20	27.3	Ins	8.5	/	/

37	1	p.N1244D	het	14	/	37	30	/	9.9	/	/
38	1	p.V1523L	het	36		46	26.5	OAD	6.5	/	/
39	1	p.Q485R	het	13	/	19	26.6	OAD	6.2	/	/
40	1	p.E100K	het	42	/	46	25	OAD and insulin	6.5	/	/
41	1	p.E1506K	het	/	/	/	/	/	/	/	/
41	2	p.E1506K	het	/	/	/	/	/	/	/	/
41	3	p.E1506K	het	/	/	/	/	/	/	/	/
41	4	p.·E1506K	het	/	/	/	/	/	/	/	/
41	5	p.E1506K	het	/	/	/	/	/	/	/	/
41	6	p.E1506K	het	/	/	/	/	/	/	/	/
41	7	p.E1506K	het	/	/	/	/	/	/	/	/
41	8	p.E1506K	het	/	/	/	/	/	/	/	/
41	9	p.E1506K	het	/	/	/	/	/	/	/	/
41	10	p.E1506K	het	/	/	/	/	/	/	/	/
42	1	p.A1536V	het	29	/	30	26.2	Dietary	/	/	/
43	1	p.G1478R	het	30	/	44	33	Insulin	/	/	/
44	1	p.Q1458E	het	21	/	30	23	Insulin	/	/	/
45	1	p.R1538Q	het	47	/	72	23.7	OAD	/	/	/
45	2	p.R1538Q	het	39	/	40	42.2	OAD	/	/	/
46	1	p.L1430F	het	20	/	43	24.9	Insulin	/	/	/
47	1	p.A1507P	het	50	/	65	28.4	Insulin	/	/	/
47	2	p.A1507P	het	38	/	40	31.1	OAD	/	/	/

48	1	p.A1390V	het	39	/	/	/	OAD	/	/	/
48	2	p.A1390V	het	20	/	/	/	Dietary	/	/	/
48	3	p.A1390V	het	50	/	56	/	OAD, insulin	/	/	/
48	4	p.A1390V	het	38	/	/	/	Insulin	/	/	/
49	1	p.R306H	het	27	19	/	/	OAD	7.2	/	/
49	2	p.R306H	het	64	/	/	/	/	/	/	/
50	1	p.L171F	hom	27	17.8	13	/	OAD	12.1	14.2	/
50	2	p.L171F	het	30	/	37	/	OAD, insulin	10.4	16.6	/
50	3	p.L171F	het	13	/	40	/	Insulin	/	/	/
51	1	p.V607M	het	/	22.9	37	/	Insulin	12.6	14.4	/
51	2	p.V607M	het	9	24.6	65	/	Insulin	12.0	16.7	/
51	3	p.V607M	het	/	19.8	40	/	Insulin	/	/	/
52	1	p.V84I	het	32	/	19	18.1	/	5.9	6	8.4
52	2	p.V84I	het	14	/	19	17.8	/	6.2	6.2	/
52	3	p.V84I	het	35	/	15	20	/	6.1	6.1	/
52	4	p.V84I	het	19	/	41	24.3	Dietary	5.9	6	/
52	5	p.V84I	het	12	/	32	21	Dietary	6.3	6.1	/
52	6	p.V84I	het	12	/	59	/	/	/	6.8	/
53	1	p.C435R	het	13	/	38	21	Insulin	6.2	/	/
54	1	p.R1379H	het	20	/	64	27	Insulin	7.4	/	/
54	2	p.R1379H	het	20	/	63	27	OAD, GLP1-RA	6.9	/	/
55	1	p.R825W	het	18	/	18	25.8	OAD, insulin	12.3	/	/
55	2	p.R825W	het	55	/	68	22.9	OAD	8.1	/	/
55	3	p.R825W	het	51	/	66	30	OAD	7.8	/	/
55	4	p.R825W	het	60	/	72	22.8	OAD	7	/	/

55	5	p.R825W	het	/	/	60	/	Insulin	/	/	/
55	6	p.R825W	het	27	/	49	27.1	Insulin	11.3	/	/
56	1	p.F793Sfs71+c·4608+4A>G	het	32	/	33	25.3	Insulin	7.9	7.1	/
57	1	p.A1536T	het	10	/	33	25.3	OAD	9.4	12.4	/
58	1	p.F1067I	het	3	/	/	/	Insulin	/	20.6	/
59	1	p.R1379H	het	/	/	/	/	/	/	/	/
60	1	p.M1513T	het	10	/	/	/	/	/	5.7	/
60	2	p.M1513T	het	/	/	/	/	OAD	/	/	/
61	1	p.S1385F	het	33	/	/	/	/	/	/	/
61	2	p.S1385F	het	/	/	/	/	/	/	/	/
62	1	p.E1205K	het	24	/	/	/	/	/	/	/
62	2	p.E1205K	het	/	/	/	/	/	/	/	/
63	1	p.R1352H	het	18	/	/	/	/	/	/	/
63	2	p.R1352H	het	/	/	/	/	/	/	/	/
64	1	p.A355T	het	/	/	/	/	/	/	/	/
64	2	p.A355T	Het	/	/	/	/	/	/	/	/
65	1	p.S53C	het	30	/	39	/	Diet	/	/	/
66	1	p.A235T	het	34	/	53	/	Insulin	/	/	/
67	1	p.V563D	het	12	/	50	/	Insulin	/	/	/
68	1	p.G658V	het	12	/	43	/	Insulin	/	/	/
69	1	p.D673N	het	14	/	27	/	OAD	/	/	/
70	1	p.R825W	het	21	/	52	/	OAD	/	/	/
71	1	p.L1147R	het	17	/	51	/	Insulin	/	/	/
72	1	p.K1384Q	het	30	18.6	45	20.8	OAD and insulin	11.9	7.5	/
72	2	p.K1384Q	het	12	20.1	/		No	/	/	/

72	3	p.K1384Q	het	14	22.8	/	/	No	/	/	/
72	4	p.K1384Q	het	55	/	/	/	No	/	/	/
73	1	p.G832S	het	42	30.3	/	/	Diet	5.9	/	/
73	2	p.G832S	het	25	21.4	/	/	OAD	5.9	/	/
73	3	p.G832S	het	50	23.5	/	/	OAD	7.1	/	/
73	4	p.G832S	het	23	24.6	/	/	OAD	6.6	/	/
74	1	p.Q833K	het	23	16.2	/	/	OAD	5.3	/	/
74	2	p.Q833K	het	18	21.6	/	/	OAD	7.4	/	/
74	3	p.Q833K	het	40	30.1	/	/	Diet	6.5	/	/
74	4	p.Q833K	het	52	20.2	/	/	OAD	4.9	/	/
75	1	p.E1506K	het	27	17.6	/	/	Diet	6.5	/	/
76	1	p.E1506K	het	26	/	/	/	Ins	10.1	/	/
77	1	p.P1198L	het	27	/	75	20.8	OAD	/	/	3.0
77	2	p.P1198L	het	41	/	41	22.8	OAD	6.7	/	3.9
77	3	p.P1198L	het	11	/	18	27.1	No	6.6	/	/
78	1	p.R168C + p.R1420C	CH	15	/	31	25.7	OAD	6.7	/	/
78	2	p.R168C + p.R1420C	CH	18	/	28	22.1	OAD and insulin	7.8	/	/
79	1	p.H863R	het	/	/	/	/	/	/	/	/
80	1	p.M1504T	het	/	/	/	/	/	/	/	/
81	1	p.A269D	het	/	/	/	/	/	/	/	/
82	1	p.R1182W	het	/	/	/	/	/	/	/	/
83	1	p.R1182Q	het	/	/	/	/	/	/	/	/
84	1	p.R1379H	het	/	/	/	/	/	/	/	/
85	1	p.R1352H	het	17	22.7	17	/	OAD and insulin	6.7	/	/

85	2	p.R1352H	het	/	/	45	/	/	/	/	/
86	1	p.G1478R	het	/	/	/	/	/	/	/	/
87	1	p.R825W	het	/	/	/	/	/	/	/	/

[#] means the number of the family. * If the age at the diagnosis of the patient was descripted with years in range, the higher limit of age was used. Abbreviations: *ABCC8*-NNDM, *ABCC8* variant-induced nonneonatal diabetes mellitus. BMI, body mass index; FPG, fasting plasma glucose; FINS, fasting insulin; OAD, oral anti-diabetic drugs; GLP1-RA, GLP1- receptor agonist.

Supplement Table 2. The classification of the *ABCC8* variants causing nonneonatal diabetes mellitus in previous publications according to the the standards and guidelines recommended by American College of Medical Genetics (ACMG)

No.	Amino acid change	Evidence of pathogenicity	ACMG Classification	The subtypes of *ABCC8*-NNDM
1	p.N780S	PM1PM2PP3PP4	likely pathogenic	uncertain
2	p.G1008S	PM1PM2PP3PP4	likely pathogenic	uncertain
3	p.A1472T	PM1PM2	uncertain significance	uncertain
4	p.E970V	PM1PM2PP3PP4	likely pathogenic	uncertain
5	p.K1022Q	PM1PM2PP3PP4	likely pathogenic	uncertain
6	p.G296R	PM1PM2PP3PP4	likely pathogenic	active
7	p.Y356C	PS3PM1PM2PP1PP3PP4	pathogenic	active
8	p.R825W	PS3PM1PM2PM5PP1PP3PP4	pathogenic	active
9	p.R1379H	PM1PM2PM5PP3PP4	likely pathogenic	active
10	p.E1506K	PS3PM1PM2PM5PP3PP4	pathogenic	inactive
11	p.R825Q	PM1PM2PM5PP3PP4	likely pathogenic	uncertain
12	p.R306C	PM1PM2PM5PP1PP3PP4	likely pathogenic	inactive
13	p.H103Y	PM1PM2PP3PP4	likely pathogenic	uncertain
14	p.R74Q	PM1PM2PP3PP4	likely pathogenic	inactive
15	p.R1493G	PM1PM2PM5PP3PP4	likely pathogenic	uncertain
16	p.A1457T	PM1PM2PP1PP3PP4PP5	likely pathogenic	inactive
17	p.R1420H	PS3PM1PM2PM5PP3PP4	pathogenic	inactive
18	p.R1418H	PS3PM1PM2PP1PP3PP4	pathogenic	inactive
19	p.P201S	PM1PM2PP3PP4	likely pathogenic	uncertain
20	p.C418R	PM1PM2PP3PP4	likely pathogenic	inactive
21	p.R620C	PM1PP3	uncertain significance	inactive
22	p.C435R	PM1PM2PP3PP4	likely pathogenic	active
23	p.L582V	PS3PM1PM2PP1PP3PP4	pathogenic	active

24	p.F591fs604X	PVS1PM2PM3PP4	pathogenic	inactive
25	p.R370S	PS3PM1PM2PP3PP4	pathogenic	active
26	p.R933X	PVS1PM2PM4PP3PP4	pathogenic	inactive
27	p.L1191LfsX1207	PVS1PM2PM4PP4	pathogenic	inactive
28	p.R1250X	PVS1PM2PM4PP3PP4	pathogenic	inactive
29	p.L1147R	PM1PM2PP3PP4	likely pathogenic	uncertain
30	p.G214R	PM1PM2PP3PP4	likely pathogenic	active
31	p.V222M	PM1PM2PP3PP4	likely pathogenic	inactive
32	p.N1244D	PM1PM2PP3PP4	likely pathogenic	uncertain
33	p.V1523L	PM1PM2PM5PP3PP4	likely pathogenic	active
34	p.Q485R	PM1PM2PP3PP4	likely pathogenic	active
35	p.E100K	PM1PM2PP4	uncertain significance	active
36	p.A1536V	PM1PM2PM5PP1PP3PP4	likely pathogenic	uncertain
37	p.G1478R	PM1PM2PM5PP1PP3PP4	likely pathogenic	inactive
38	p.Q1458E	PM1PM2PP1PP3PP4	likely pathogenic	uncertain
39	p.R1538Q	PM1PM2PP1PP3PP4	likely pathogenic	inactive
40	p.L1430F	PM1PM2PP1PP3PP4	likely pathogenic	uncertain
41	p.A1507P	PM1PM2PP1PP3PP4	likely pathogenic	inactive
42	p.A1390V	PM1PM2PP1PP3PP4	likely pathogenic	uncertain
43	p.R306H	PM1PM2PM5PP1PP3PP4	likely pathogenic	active
44	p.L171F	PM1PM2PM5PP1PP3PP4	likely pathogenic	uncertain
45	p.V607M	PM1PM2PP1PP3PP4	likely pathogenic	active
46	p.V84I	PM1PM2PP1PP3PP4	likely pathogenic	uncertain
47	p.F793Sfs71	PVS1PM2PM4PP4	pathogenic	inactive
48	p.A1536T	PM1PM2PM5PP3PP4	likely pathogenic	uncertain
49	p.A1366T	PM2PP3PP4	uncertain significance	uncertain

50	p.F1067I	PM1PM2PP3PP4	likely pathogenic	active
51	p.M1513T	PM1PMPM5PP3PP4	likely pathogenic	uncertain
52	p.S1385F	PM1PM2PP3PP4	likely pathogenic	uncertain
53	p.E1205K	PM1PM2PP3PP4	likely pathogenic	uncertain
54	p.R1352H	PM2PP3PP4	uncertain significance	inactive
55	p.A355T	PM1PM2	uncertain significance	inactive
56	p.S53C	PM1PM2PP3PP4	likely pathogenic	uncertain
57	p.A235T	PM1PM2	uncertain significance	uncertain
58	p.V563D	PM1PM2PP3PP4	likely pathogenic	uncertain
59	p.G658V	PM1PM2PP4	uncertain significance	uncertain
60	p.D673N	PM1PM2PP4	uncertain significance	uncertain
61	p.K1384Q	PM1PM2PM5PP1PP3PP4	likely pathogenic	inactive
62	p.G832S	PM1PM2PM5PP1PP3PP4	likely pathogenic	uncertain
63	p.Q833K	PM1PM2PP1PP3PP4	likely pathogenic	uncertain
64	p.P1198L	PS3PM1PM2PP1PP3PP4	pathogenic	active
65	p.R168C	PM1PM2PP1PP3PP4	likely pathogenic	inactive
66	p.R1420C	PM1PM2PM5PP1PP3PP4	likely pathogenic	inactive
67	p.H862R	PM1PM2PM5PP1PP3	likely pathogenic	uncertain
68	p.M1504T	PM1PM2PP3PP4	likely pathogenic	uncertain
69	p.A269D	PM1PM2PP3PP4	likely pathogenic	active
70	p.R1182W	PS3PM1PM2PM5PP3PP4	pathogenic	active
71	p.R1182Q	PS3PM1PM2PM5PP1PP3PP4	pathogenic	active

Abbreviations: *ABCC8*-NNDM, indicates *ABCC8* variant-induced nonneonatal diabetes mellitus. PM, pathogenic moderate; PP, pathogenic supporting; PS, pathogenic strong; PVS, pathogenic very strong.

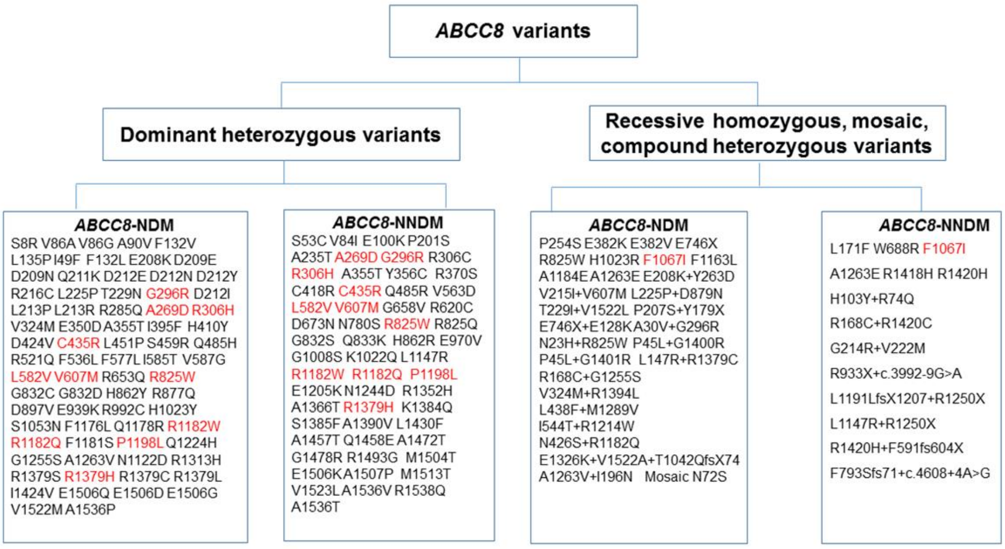

Supplement Figure 1. A diagram illustrates the inheritance of *ABCC8* variants in probands with neonatal diabetes mellitus and nonneonatal diabetes mellitus. Variants showed in red represent the variants were identified both in neonatal diabetes mellitus and nonneonatal diabetes mellitus.

Abbreviations: *ABCC8*-NDM, *ABCC8* variant-induced neonatal diabetes mellit*us*; *ABCC8*-NNDM, *ABCC8* variant-induced nonneonatal diabetes mellitus.
